# Opioid Prescription Patterns for Discharged Patients from the Emergency Department

**DOI:** 10.1155/2021/4980170

**Published:** 2021-01-13

**Authors:** Justin Yanuck, Jonathan B. Lee, Soheil Saadat, Jila Rouhi, Ghadi Ghanem, Bharath Chakravarthy, Shalini Shah

**Affiliations:** ^1^Department of Anesthesia, Critical Care and Pain Medicine, Massachusetts General Hospital, 55 Fruit Street, Boston 02114, Massachusetts, USA; ^2^Department of Emergency Medicine, University of California, Irvine, California, USA; ^3^Department of Anesthesiology & Perioperative Care, University of California, Irvine, California, USA

## Abstract

**Objectives:**

It is important to analyze the types of etiologies and provider demographics that drive opioid prescription in our emergency departments. Our study aimed to determine which patients in the ED are receiving opioid prescriptions, as well as their strength and quantity. Secondary outcomes included identifying difference in prescribing between provider classes.

**Methods:**

We conducted a retrospective study at a tertiary care university-based, level-one trauma ED from November 2017 to October 2018. We identified and analyzed data from 2,259 patients who were sent home with an opioid prescription. We retrieved patient and provider demographics, diagnosis, etiologies, and prescription information.

**Results:**

The mean age of a patient receiving an opioid prescription was 45, and 72.7% of patients were white. The most common diagnosis groups associated with an opioid prescription were abdominal pain (18.5%), nonfracture extremity pain (18.4%), and back/neck pain (12.5%). Hydrocodone-acetaminophen 5–325 mg was the most commonly prescribed (67.4%). The median total prescribed milligram morphine equivalent (MME) was highest for extremity fracture (75.0; IQR 54.0–100.0). The median total prescribed amount of pills was highest for patients with extremity fractures (15.0; IQR 12.0–20.0).

**Conclusions:**

Our study elucidates the prescribing patterns of an academic level 1 trauma center and should pave the way for future studies looking to maximize effectiveness at ways to curb ED opioid prescription.

## 1. Introduction

In 2008, prescription opioid deaths surpassed those caused by heroin [1]. This rise in prescription opioid use largely stemmed from regulatory agencies mandating that physicians treat pain as the ‘fifth' vital sign [2, 3]. Prescription opioid sales have quadrupled from 1999 to 2014; however, there is no clear evidence that the amount of pain Americans report is changing [4, 5]. In 2016, US doctors wrote 66.5 opioid prescriptions per 100 persons, and an estimated 4.7 per 100 persons misused prescription opioids [6]. Given that over 60% of patients present to the ED with a complaint of pain, it logically follows that opioid prescriptions in the ED have gradually increased [7]. Understanding the “how” of this increase is equally important to track and trend in addition to tracking the increase in opioid output.

A recent observational study showed that for every 48 patients prescribed a new opioid in the ED, one patient will become a long-term opioid user [8]. While this study cannot ascribe causality, other studies have also supported this trend [9, 10]. For years, many believed that a transient encounter in the ED would be unlikely to lead to long-term opioid abuse. However, it has been observed that in nearly 50% of patients encountered in the ED who reported heroin or prescription opioid abuse, their first exposure to opioids was from an ED prescription [10]. Although these data are far from conclusive, it hints at a trend that is deeply concerning.

Recently, there has been a push to curtail physician opioid prescribing patterns. These efforts, for the most part, have largely been based on limiting the number of opioids prescribed from the ED to short courses, identifying patients at risk for abuse, and identifying physicians that prescribe the most. One multicenter study was conducted in 2012 over a week-long period that analyzed the various diagnoses in the ED that were prescribed opioids. The results indicate a variety of diagnoses prescribed opioids, with musculoskeletal back pain and abdominal pain being the largest proportion of patients discharged with an opioid prescription [11]. However, this study was limited to one week of data. Furthermore, this study does not assess for how recent opioid prescribing guidelines as well as an increased awareness of the opioid epidemic has affected opioid prescribing patterns over the last 5 years [12–15].

For context, in California, providers must consult the Controlled Substance Utilization Review and Evaluation System (CURES), which is the state's prescription drug monitoring program, prior to prescribing Schedules II-IV controlled substances for the first time and at least once every four months thereafter if the patient continues to use controlled substances. However, if these medications are prescribed within the emergency department, providers do not have to consult CURES if the quantity of controlled substance does not exceed a nonrefillable seven day supply [16]. The general practice at this institution is to prescribe less than 1 week of opioids, and thus most prescriptions are exempt from CURES. Individual providers check CURES base on their personal preferences and based on suspicion for abuse potential.

There is an immediate need for the development of targeted, nonopioid pain therapy for common diagnoses in the ED. In this study, we aim to further analyze which diagnoses (ICD-10) are receiving opioids as well as characterize both the patients who are receiving these opioids and the providers who are prescribing. With this information, we hope to add to the existing data to create directed pain protocols for a fixed diagnosis more confidently.

## 2. Methods

### 2.1. Study Design and Settings

We conducted a retrospective chart review study at a tertiary, academic level-one trauma center, university-based ED between November 2017 to October 2018.

### 2.2. Selection of Participants

Patients aged 18 or older that presented to the ED and were discharged with an opioid medication between the study period were analyzed. We excluded patients who were admitted to the hospital, transferred to another hospital, or not discharged with a new opioid prescription. The study was reviewed and approved by the university's Institutional Review Board as an exempt category. Patient informed consent was not applicable to this study.

### 2.3. Measurements

Our data were obtained from the hospital medical record database. We extracted the following information for each patient from this database: age, sex, ethnicity, insurance payor, provider (specialty; physician versus nurse practioner; and years in practice), diagnosis (ICD-10), prescription medication (name and dose), and quantity (number of tablets). For each patient, a total prescribed milligram morphine equivalent (MME) was calculated by multiplying the prescribed amount (in mg) by potency of prescribed medication. In this study, the total MME, prescribed amount, and number of pills prescribed refer only to medications prescribed to patients upon discharge and does not include any medications administered within the ED visit. We grouped each patient diagnosis into one of the following etiological categories: abdominal pain, nonfracture extremity pain, extremity fracture, headache, chest pain, genitourinary, back/neck pain, unspecified trauma, dental/facial pain, and others/missing/unspecified. The primary outcome of this study was to identify chief complaints associated with the highest opioid prescriptions upon discharge in terms of total MME and total amount (number of pills). Secondary outcomes included differences in the total MME and total number of pills prescribed upon discharge amongst different provider classes (EM attending physicians, resident physicians, nurse practitioners, and consulting physicians) along with identifying the most commonly prescribed opioid formulations (hydrocodone, morphine, codeine, etc.).

### 2.4. Analysis

Frequencies are presented as *N* (%) and continuous variables as mean (SD) or median (IQR), according to their distribution. The KolmogorovSmirnov test was used to examine the distribution of continuous variables. The independent-sample median test was used to compare total prescribed MME and also number of pills prescribed between EM vs. non-EM providers. A *P* value <0.05 was considered statistically significant. IBM SPSS Statistics 26 for windows was used for data analysis.

## 3. Results

### 3.1. Characteristics of Study Subjects

From November 2017 to October 2018, a total of 2,259 unique records were obtained retrospectively from visits associated with an opioid prescription upon discharge from the ED. Of these, mean age was 44.86 (SD 16.84), and 73.0% (*N* = 1648) of patients were white, 9.3% (*N* = 209) asian, and 4.4% (*N* = 99) black. Furthermore, 49.2% (*N* = 1111) of these patients had Medicaid insurance, 25.8% (*N* = 583) had a commercial insurance, and 14.5% (*N* = 328) had Medicare (Table 1).

### 3.2. Baseline Characteristics of the Study Population

#### 3.2.1. Main Results

**Figure 1:**
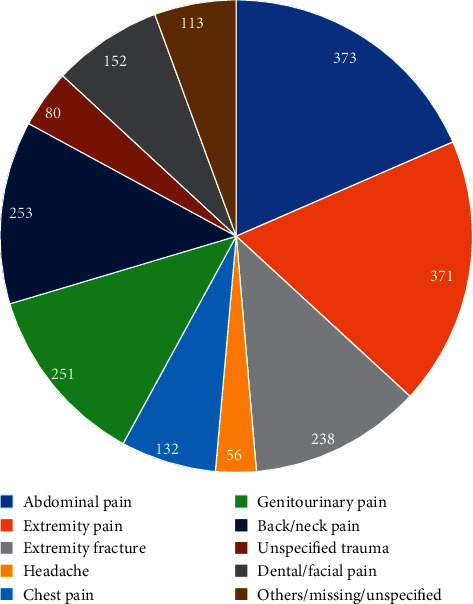
Opioid prescriptions by body system.

Within the study time frame, 54,190 patients were seen in total, 14,177 (26.2%) patients were admitted to the hospital, and 40,013 (73.8%) patients were discharged from the emergency department. Given there were 2,259 patients included in this study, only 4.2% of patients who were discharged from the emergency department received a prescription for an opioid. Of these patients, the most common diagnosis groups associated with an opioid prescription were abdominal pain (16.5%, *N* = 373), nonfracture extremity pain (16.4%, *N* = 371), back/neck pain (11.2%, *N* = 253), genitourinary (11.1%, *N* = 251), and extremity fracture (10.5%, *N* = 238) (Figure 1).

The median total prescribed milligram morphine equivalent (MME) was highest for extremity fracture (75.0; IQR 54.0–100.0), and for the category of “others/missing/unspecified” (75.0; IQR 50.0–100.0), all other body system categories had a median of 60.00 with varying IQR (Table 2). The median total prescribed amount of pills was highest for patients with extremity fractures (15.0; IQR 12.0–20.0) and the body systems associated with the least amount of pills prescribed were abdominal pain (10.0; IQR 9.0–15.0) and unspecified trauma (10.0; IQR 8.0–15.0) (Table 2).

Hydrocodone-acetaminophen 5–325 mg was the most commonly prescribed (67.4%, *N* = 1523), followed by hydrocodone-acetaminophen 10–325 (8.9%, *N* = 200), tramadol 50 mg (7.6%, *N* = 171), acetaminophen-codeine #3 300–30 mg Tabs (3.5%, *N* = 78), oxycodone-acetaminophen 5–325 mg tabs (2.9%, *N* = 65), oxycodone 5 mg tabs, oxycodone-acetaminophen 10–325 mg tabs (1.3%, *N* = 29), and morphine sulfate 15 mg tabs (1.0%, *N* = 22). All other opioid prescriptions were prescribed at less than 1% frequency and are not listed. Of note, all of the above listed medications were immediate release formulations. Extended release formulations of opioids were all prescribed at less than 1% frequency and thus not listed above.

**Figure 2:**
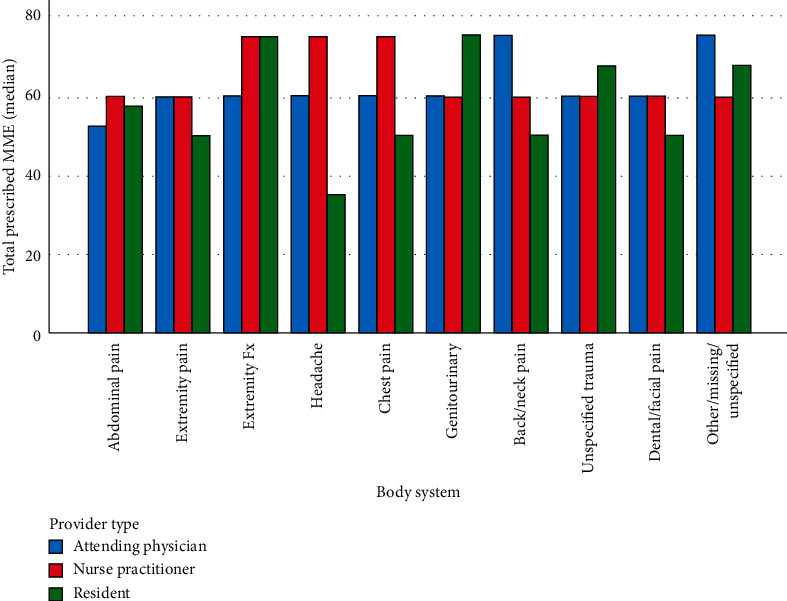
Subgroup analysis-total MME. This subgroup analysis depicts how each provider type (attending physician, nurse practitioner, and resident physician) prescribes.

**Figure 3:**
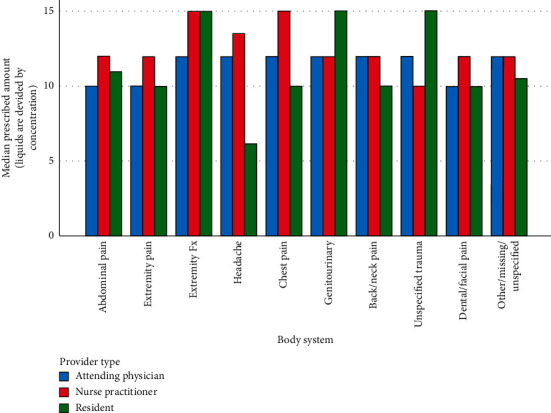
Subgroup analysis-total number of pills. This subgroup analysis depicts how each provider type (attending physician, nurse practitioner, and resident physician) prescribes. Given some formulations were prescribed as liquids, these corresponding number of pills are derived from the concentration.

When analyzing opioid prescriptions based upon the provider class (attending physician, resident physician, or nurse practitioner), 47.1% (*N* = 1065) of the prescriptions were made by attending physicians, 32.6% (*N* = 737) by nurse practitioners, and 20.2% (*N* = 457) by resident physicians. A subgroup analysis revealed no statistically significant difference in the provider classes prescribing habits, for both total MME (*P*=0.211) and for total number of pills prescribed (*P*=0.456) (Figures 2 and 3)

Furthermore, an analysis was performed and it was found that a total of 5.0% of prescriptions were made by non-EM providers. Non-EM providers tended to prescribe more than EM providers in terms of median total MME (60.0 vs 12.0) (*P* < 0.001) and median number of pills prescribed (100.0 vs. 20.0) (*P* < 0.001) (Table 3). Additionally, a supplementary analysis was performed to determine if prescriber demographics such as gender or experience level (estimated by the number of years postgraduation from professional school) had any association with prescribing habits such as total MME prescribed and total number of pills prescribed (see supplementary figure 1 in the Appendix). However, there was no evidence of an association when examining the data.

This table represents an analysis in which emergency medicine (EM) providers were compared to non-emergency medicine (Non-EM) providers in terms of total MME and total number of pills, represented in medians.

Also found was the frequency of instances in which providers prescribed opioids in the presence of an already existing opioid prescription as seen in Table 4. Given there were 2,259 patients in the study, we can conclude that only 9.5% (*n* = 214) of opioids were prescribed in the presence of an already existing opioid prescription. Of these 214 cases, 30.4% of the total MME was increased, 39.4% of the time, it was decreased, and 30.2% of the time, it was kept equal. For 41.1% of the time, the provider discontinued the prior opioid prescription, whereas 58.9% of the time prior prescriptions were unchanged and an opioid was added.

## 4. Limitations

Given our study was conducted at a single, level-one trauma center, university-based ED, our data may not be generalizable to other populations or other emergency departments. Higher acuity patients inherent to a level-one trauma center, social and medical complexity of the patient population, and timely access to primary care are all factors that may limit the external validity of this study.

Another limitation to this study is that diagnosis codes were not available for each patient. Of the 2259 patients included in the study, 240 (10.6%) had missing diagnosis codes and 113 (4.8%) had diagnosis codes that could not be classified into a body system given the ambiguity of certain ICD codes. Furthermore, the diagnoses were assigned during or shortly after an ED encounter (verses after hospitalization, additional imaging, or outpatient specialist follow up). Thus, diagnosis may lack specificity or may not reflect the true diagnosis of the patient.

Furthermore, our study had a few outliers. A common scenario that explained our upper limit outliers were physicians from the palliative care service prescribing large quantities and higher MMEs to opioid tolerant patients. Although these prescriptions were not made by ED trained physicians or NPs, they were still made upon discharge from the ED by the consulting service and were thus included within the analysis. In order to limit the effects of these outliers, medians were used instead of means for the majority of our analyses. Although only 5% of opioids prescribed were from non-EM providers, our study found that non-EM-trained providers prescribed a statistically significant greater amount of opioids in terms of both total MME and total number of pills. Therefore, this study can only claim to depict prescription habits from the emergency department as a whole not solely from EM-trained faculty.

Our subgroup analysis may have been limited, given NPs in this particular ED are assigned lower acuity patients and are seen in the ED's “Fast-Track,” whereas ED attendants and residents may see both low- and high-acuity patients. A difference in patient population and severity of illness therefore may have confounded the results of the subgroup analysis. Additional retrospective observational studies looking into attending, residents, and nurse practitioner prescribing habits for low-acuity patients (Emergency Severity Index: 4–5) may be required to correct for confounding variables present in this study.

Lastly, given analysis was based mainly off of diagnosis codes, we are unable to ascertain how the severity of each condition might be related to the prescription of opioids. At this particular institute, pain scores are not consistently recorded by physician or nursing staff, and therefore given the retrospective nature of this study, we are unable to ascertain the level of severity of each injury. Although some diagnoses can be inferred to appropriately warrant opioid pain prescriptions (i.e., open fracture), there are challenges to determining the appropriateness of opioid pain medications for many other diagnoses (i.e., abdominal pain and headache) without factors such as severity or duration of illness.

## 5. Discussion

This medical center is located in Orange County, California, which is a densely populated setting in Southern California. Orange county is ranked 17^th^ out of 58 counties in California for rates of prescription opioid deaths and unintentional injuries, drug overdose being the largest contributor, which was the number 1 cause of death in patients between the ages of 15 and 44 [17, 18].

Rising trends in ED opioid prescriptions is a public health concern, given studies have demonstrated the potential for these medications to be misused, diverted, or lead to opioid related deaths [19, 20]. The data within this study elucidate recent trends in opioid prescriptions in relation to common diagnoses associated with opioid prescriptions, amount and type of opioid prescribed, and provider type (i.e., attending physician, NP/PA, and resident).

Similar to the prior cross-sectional study, this study demonstrates that the most common diagnoses associated with opioid pain prescriptions were extremity pain, abdominal pain, and back/neck pain [11]. Identifying which diagnosis is associated with higher frequency of opioid prescriptions can be crucial to implementing symptom specific guidelines for opioid prescriptions in an effort to curtail the use of opioids in the ED and upon discharge. A previous study demonstrated a reduction of opioid administration for renal colic after implementation of a opioid-reduction initiative based on pain syndrome-targeted opioid alternative protocols [21]. Similarly, a number of recent studies have demonstrated a reduction in opioid prescriptions after the implementation of opioid prescribing guidelines for emergency physicians [22, 23].

In order to maximize the effect of the implementation of such guidelines, targeting patients with extremity pain, abdominal pain, and back/neck pain may be the best initial steps to reducing opioid prescriptions as a whole given these diagnoses are more frequently associated with opioid pain medications. Combining extremity pain, extremity fracture, and back/neck pain under a single “Musculoskeletal” category would account for 42.7% of the opioid prescriptions. Thus, exploring specific approach to musculoskeletal pain may account for nearly half of the patients in this particular center. In order to implement more targeted prescribing guidelines, additional analysis can be performed in order to divide the broad diagnosis groups (i.e., abdominal pain) presented in this study into more specific diagnoses (i.e., biliary colic and diverticulitis).

In our study, extremity fracture pain was associated with the highest median number of pills dispensed at 15.0 pills, with most other diagnosis groups receiving less than 12 pills total. This is consistent with available guidelines which suggest no more than three days worth of opioids for acute pain assuming six times daily dosing regimens [24, 25]. Also, consistent with these available guidelines, our data show that the vast majority of prescriptions are written in the lower dose ranges and in immediate release formulations. For example, hydrocodone-acetaminophen 5–325 mg (67.4%) makes up the majority of our opioid prescriptions compared to the higher dosage formulates of 7.5–325 mg (0.3%) and 10–325 mg (8.9%). One exception to this observation is that we observe acetaminophen-codeine #3 300–30 mg (3.5%) being prescribed more frequently than acetaminophen-codeine #2 300–15 mg (0.0%). Given these medications combined make up only 3.5% of opioid prescriptions, this finding is the exception to the overall trend of preferential prescribing at lower doses. Given this study was performed at a single academic center, the opioid usage patterns may be influenced by increased awareness of the opioid crisis or even attending physician's desire to influence and foster resident physicians' future opioid prescribing habits. The patients with extremity pain received a median of 12 (IQR 10–15)) and total MME median of 60.00 (IQR 50–100). In another study looking into ankle sprains across multiple regional EDs, it was found that the median number of pills to be 15 (IQR 12–20) and medial total MME of 100 (IQR 75–113) [26]. Although the data are not entirely comparable, given our data on extremity pain are not limited to ankle sprains, it points towards a more conservative opioid usage pattern in our study population.

A total of 1.3% of ED opioid prescriptions are written for extended release or long acting (ER/LA) formulations which include methadone (0.5%), morphine ER (0.5%), and oxycodone ER (0.3%). These habits are consistent with CDC guidelines suggesting immediate-release opioid formulations given ER/LA opioids have been associated with greater risk for overdose [26, 27]. One potential explanation for the prescriptions of ER/LA formulations are patients presenting with existing prescriptions that were renewed by the emergency physician within the ED encounter. Another possibility is the use of these extended release formulations, methadone in particular, as a means to treat opioid use disorder rather than an acute cause of pain.

Hydrocodone-containing products (77.4%), oxycodone-containing products (8.9%), and morphine (1.6%) were found to be prescribed in decreasing frequency as listed above. A systematic review of nine studies comparing abuse potential of these three products found that oral oxycodone had an elevated abuse liability profile when compared to morphine and hydrocodone [28]. Furthermore, a study comparing crowdsourcing black market prices for prescription opioids found the following street values of opioids when diverted: oxycodone (US$0.97), hydrocodone (US$.81), and morphine (US$0.52) [29]. Guidelines to be designed and implemented for ED providers should take into account prescribing opioids with decreased abuse potential (hydrocodone and morphine) and decreased street value (morphine > hydrocodone > oxycodone) in order to reduce the risk of dependency, misuse, and diversion. Future survey-based cross-sectional studies on provider preferences for PO opioid prescriptions and provider's prior knowledge of abuse potential and/or street value opioids may be helpful in elucidating medical decision-making behind prescription patterns.

The fact that no significant difference was found for prescribing habits between the three provider classes (attendings, residents, and NPs) seems to corroborate the findings of a previous study that showed that patient encounters involving physician trainees reflected similar trends in attending practice. [30] At this particular institution, 2^nd^ and 3^rd^ year resident physicians obtain licenses to prescribe opioids. In general, the decision to prescribe opioids, both type and quantity of pills, is left up to the discretion of the resident prescribing with minimal attending physician input after the first year of training. The same is true for nurse practitioners, who work fairly independently at this particular institution and use their own license to prescribe opioids. Despite the lack of statistical significance, there are several notable trends. Residents tended to have more variability in their prescribing habits across different organ systems, whereas attending physicians and nurse practitioners tended to have more consistency when prescribing across different organ systems. For unspecified trauma, genitourinary, and extremity fractures, resident physicians tended to prescribe the most number of pills and overall higher amounts of MME. However, for headaches, resident physicians seemed to have prescribed less than any other provider class for both MME and number of pills. Although the reasoning cannot be ascertained from the data alone, one possible explanation could be that the resident physicians have not had enough time to develop their particular practice patterns.

Furthermore, the fact that residents seemed to prescribe opioids for headache notably less than any other chief complaint may be attributable to resident physician training, which includes preparation for EM in training examinations and eventually EM board certification upon graduating from residency. Common resources used in studying for these examinations are Adam's Emergency Medicine, Rosen's Emergency Medicine, and Tintinalli's Emergency Medicine textbooks. In all three of these resources, it is stressed that opioids are only to be used for headaches as a second line therapy, and even then, they should be used sparingly and to be considered only if there is a lack of response and/or intolerance of all first line therapies which include but are not limited to acetaminophen, ibuprofen, prochlorperazine, metoclopramide, sumatriptan, or ketorolac. [31–33] It is possible that resident physicians are more likely to practice in more “Textbook” methods, which in this case means prescribing opioids less often for headaches in particular. Our study did not support the findings of Leventhal E. L et al. who found that EM-trained residents prescribed fewer opioids to patients upon discharge from the ED compared to EM attending physicians [34].

## 6. Conclusion

Our study found that opioid-prescribing habits (total MME, total number of pills, and low dosage/instant release formulations) were in accordance with national opioid prescribing guidelines that recommend no more than three days of opioids assuming six times daily dosing with a preference towards immediate release formulations. Given abdominal pain (18.5%), extremity pain (18.4%), and back/neck pain (12.5%) were the most common chief complaints that received opioids, any future guidelines for curbing opioid prescriptions upon discharge should have a strong emphasis on these chief complaints. Although there was no difference in prescribing habits between different provider classes (attendings, residents, or NPs), future studies accounting for patient complaint severity could help to elucidate any true differences.

## Figures and Tables

**Table 1:** Baseline characteristics.

Characteristics	Classification	Percent of patients (*n* = 2259)
Age, years (mean)	44.86	
Race	Asian	9.3
	American Indian or Alaska native	0.1
	Black or African American	4.5
	Multi-race	1.7
	Native Hawaiian or pacific Islander	0.4
	Other races	11.0
	White	73.0

Ethnicity	Hispanic	44.5
	Non-Hispanic	54.3
	Unknown	1.2

Insurance type	Commercial	22.7
	Medicaid	45.3
	Medicare	15.8
	Other	2.2
	Other public	6.4
	Self pay	7.6

Gender	Male	49.3
	Female	50.7

**Table 2:** Total prescribed MME and the number of pills by the body system.

Body system	Total MME (IQR 25%ile–75%ile)	Total number of pills (25%ile, 75%ile)
Abdominal pain	60.0 (IQR 49.5–100.0)	10.0 (IQR 9.0–15.0)
Extremity pain	60.0 (IQR 50.0–100.0)	12.0 (IQR 10.0–15.0)
Extremity fracture	75.0 (IQR 54.0–100.0)	12.0 (IQR 12.0–20.0)
Headache	60.0 (IQR 40.0–100.0)	12.0 (IQR 8.0–16.0)
Chest pain	60.0 (IQR 50.0–100.0)	12.0 (IQR 10.0–15.0)
Genitourinary	60.0 (IQR 50.0–100.0)	12.0 (IQR 10.0–16.0)
Back/neck pain	60.0 (IQR 50.0–100.0)	12.0 (IQR 10.0–20.0)
Unspecified trauma	60.0 (IQR 50.0–75.0)	12.0 (IQR 8.0–15.0)
Dental/facial pain	60.0 (IQR 50.0–100.0)	12.0 (IQR 10.0–15.0)
Others/missing/unspecified	75.0 (IQR 50.0–100.0)	12.0 (IQR 10.0–20.0)

**Table 3:** Difference is prescription habits by specialty.

Provider specialty	EM	Non-EM	*P* value
Total MME (median)	60.0 (IQR 50.0 to 100.0)	100.0 (IQR 60.0 to 168.8)	<0.001
Total number of pills (median)	12.0 (IQR 10.0 to 16.0)	20.0 (IQR 10.0 to 24.0)	<0.001

**Table 4:** Frequency of opioids prescribed prior to ED visit.

	Same MME	Increased MME	Decreased MME	Total
Prior opioid discontinued	27	28	33	88
Prior opioid unchanged	38	37	51	126

## Data Availability

The data used to support this study can be made available from the corresponding author upon request (jonathbl@uci.edu). The author will then make arrangements through the Institutional Review Board to ensure appropriate access is granted.

## Supplementary Materials

Supplementary Materials Figure 1: the *x* axis represents the year of graduation for each provider, while the *y* axis represents the total MME prescribed. Males (blue) and females (red) are separated. There is no difference in opioid prescribing habits when comparing the number of years of experience or when comparing the gender of the prescriber.
