# Supplementary material for: Opioid Prescription Patterns for Discharged Patients from the Emergency Department
Source: Pain Res Manag. 2021 Jan 13;2021:4980170. doi: 10.1155/2021/4980170 (PMC7837768; doi:10.1155/2021/4980170)
Supplement: Supplementary Materials — Figure 1: the x axis represents the year of graduation for each provider, while the y axis represents the total MME prescribed. Males (blue) and females (red) are separated. There is no difference in opioid prescribing habits when comparing the number of years of experience or when comparing the gender of the prescriber. [file 4980170.f1.docx]

**Appendix**

**Figure 1: Gender and Experience (# years since graduation) versus Total MME prescribed**

**
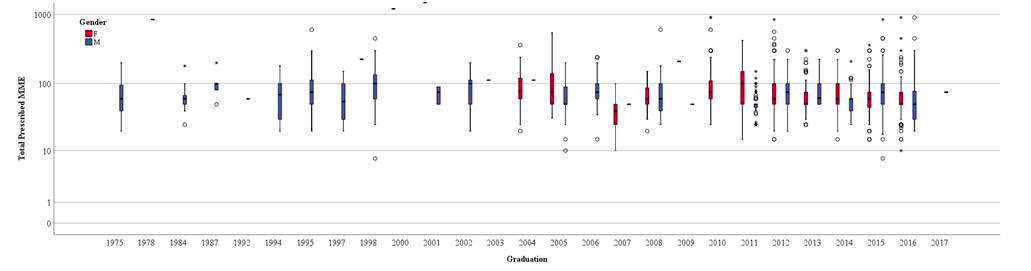
**

*On this figure, the x axis represents the year of graduation for each provider, while the y axis represents the total MME. Males(blue) and females(red) are separated.*
